# Relative abundance and molecular evolution of Lake Sinai Virus (Sinaivirus) clades

**DOI:** 10.7717/peerj.6305

**Published:** 2019-03-21

**Authors:** Robert S. Cornman

**Affiliations:** U.S. Geological Survey, Fort Collins Science Center, Fort Collins, CO, USA

**Keywords:** Lake Sinai Virus, Sinaivirus, *Apis mellifera*, *Varroa destructor*, Evolutionary rates, Codon usage

## Abstract

Lake Sinai Viruses (Sinaivirus) are commonly detected in honey bees (*Apis mellifera*) but no disease phenotypes or fitness consequences have yet been demonstrated. This viral group is genetically diverse, lacks obvious geographic structure, and multiple lineages can co-infect individual bees. While phylogenetic analyses have been performed, the molecular evolution of LSV has not been studied extensively. Here, I use LSV isolates from GenBank as well as contigs assembled from honey bee Sequence Read Archive (SRA) accessions to better understand the evolutionary history of these viruses. For each ORF, substitution rate variation, codon usage, and tests of positive selection were evaluated. Outlier regions of high or low diversity were sought with sliding window analysis and the role of recombination in creating LSV diversity was explored. Phylogenetic analysis consistently identified two large clusters of sequences that correspond to the current LSV1 and LSV2 nomenclature, however lineages sister to LSV1 were the most frequently detected in honey bee SRA accessions. Different expression levels among ORFs suggested the occurrence of subgenomic transcripts. ORF1 and RNA-dependent RNA polymerase had higher evolutionary rates than the capsid and ORF4. A hypervariable region of the ORF1 protein-coding sequence was identified that had reduced selective constraint, but a site-based model of positive selection was not significantly more likely than a neutral model for any ORF. The only significant recombination signals detected between LSV1 and LSV2 initiated within this hypervariable region, but assumptions of the test (single-frame coding and independence of substitution rate by site) were violated. LSV codon usage differed strikingly from that of honey bees and other common honey-bee viruses, suggesting LSV is not strongly co-evolved with that host. LSV codon usage was significantly correlated with that of *Varroa destructor*, however, despite the relatively weak codon bias exhibited by the latter. While codon usage between the LSV1 and LSV2 clusters was similar for three ORFs, ORF4 codon usage was uncorrelated between these clades, implying rapid divergence of codon use for this ORF only. Phylogenetic placement and relative abundance of LSV isolates reconstructed from SRA accessions suggest that detection biases may be over-representing LSV1 and LSV2 in public databases relative to their sister lineages.

## Introduction

Honey bees (*Apis mellifera*) are key pollinators of agroecosystems, yet their management has been complicated by various stressors such as transport, nutritional challenges, pesticide exposure, and pathogen pressure (Brodschneider & Crailsheim, 2010; Doublet et al., 2015). RNA viruses have been a research focus with respect to honey bee health because they underlie several defined pathologies, may be associated with colony collapse, and interact with other components of the microbiome (Chejanovsky et al., 2014; McMenamin & Genersch, 2015; Carrillo-Tripp et al., 2016). The metagenomic era has enabled a better characterization of viral diversity within honey bee hosts and accelerated the discovery of novel species (Runckel et al., 2011; Cornman et al., 2012; Ryabov et al., 2014; De Miranda et al., 2015; Gauthier et al., 2015; McMahon et al., 2016; Shi et al., 2016; Bigot et al., 2017; Remnant et al., 2017; Roberts, Anderson & Durr, 2017). The Lake Sinai Viruses (Sinaivirus) are among the most abundant of the recently described groups, uncovered by early metagenomic surveys of bee colonies in the US (Runckel et al., 2011; Cornman et al., 2012) and since identified in surveys throughout the world (Daughenbaugh et al., 2015; Ravoet et al., 2015; Roberts, Anderson & Durr, 2017). While full genome references and amplicon surveys have been published (Daughenbaugh et al., 2015; Ravoet et al., 2015; Shi et al., 2016; Bigot et al., 2017; Remnant et al., 2017; Roberts, Anderson & Durr, 2017) and phylogenetic analyses of environmental isolates have been performed, ecological relationships among lineages are not understood and their nomenclature has developed ad hoc. However, it is clear that well-differentiated LSV lineages exist that lack obvious geographic structure and can co-infect at the colony and individual levels (Ravoet et al., 2015; Bigot et al., 2017; Roberts, Anderson & Durr, 2017).

Phylogenetic analyses to date have been based partly or fully on the RNA-dependent RNA polymerase (RDRP) ORF (Daughenbaugh et al., 2015; Ravoet et al., 2015; Bigot et al., 2017; Roberts, Anderson & Durr, 2017), and have recovered two common phylogenetic clusters, termed LSV1 and LSV2, plus additional lineages that have been given other LSV labels. Small structural differences have been identified among these genomes, such as varying levels of ORF overlap, and amino-acid identities are typically 70–90%, with the capsid sequence slightly more diverged (Ravoet et al., 2015; Bigot et al., 2017). Bigot et al. (2017) used a region of ORF1 and RDRP to identify four clades they designated A–D. However, it is not clear whether phylogenetic clusters of LSV sequence reflect divergence under natural selection, demographic expansion, or perhaps some sampling bias (e.g., oversampling or primer-mediated PCR detection biases). The relative abundance of sequences attributable to different LSV clades also remains to be investigated. While coinfection has been demonstrated, it has not been tested whether sister lineages may be recombinants of LSV1 and LSV2. Single parameter estimates of evolutionary rate have been estimated for LSV ORFs (Bigot et al., 2017), but more realistic models allowing site variation have not been evaluated, nor has it been determined whether LSV codon usage is co-evolved with honey bees as is evident for other RNA viruses of this host (Chantawannakul & Cutler, 2008).

In this study, I use public sequence data to construct alignments for a large sample of each LSV ORF to investigate patterns of molecular evolution. I evaluate whether phylogenetic signal from the 3′ ORFs (capsid and ORF4) agrees with that of the 5′ ORFs (ORF1 and RDRP). I evaluate codon usage relative to that of honey bee and also Varroa mite (*Varroa destructor*), a common parasite of honey bees that is known to vector other RNA viruses (Gisder, Aumeier & Genersch, 2009; De Miranda & Genersch, 2010; Dainat et al., 2012) and may be a host of LSV as well although replication has not been demonstrated molecularly (Daughenbaugh et al., 2015). For each ORF, I compute pairwise protein divergence within sliding windows and estimate codon-specific rates of substitution; these measures of rate variation can signal genomic regions of functional or evolutionary significance. I also test for recombination between LSV1 and LSV2 lineages, which could contribute to the intermediate phylogenetic position of some lineages. Finally, I investigate the distribution of major LSV lineages in Sequence Read Archive (SRA) accessions by mapping reads to LSV1, LSV2, and their sister clades.

## Materials and methods

### Discovery of LSV sequence

Annotated ORFs from the reference LSV genome (NC_032433.1; Shi et al., 2016) were used as seeds to identify approximately full-length ORFs in the Nucleotide (nt) database of NCBI using the BLAST webserver. Well-conserved RDRP protein fragments were then used as a reference database against which *A. mellifera* and *V. destructor* accessions were searched, in order to identify and reconstruct additional LSV isolates from shotgun transcriptomic sequence. This metagenomic approach increased the sample of LSV isolates for analysis and is also potentially less biased than primer-based discovery methods. Studies that generated LSV accessions used in this study include Jamnikar-Ciglenecki, Toplak & Kuhar (2018), Runckel et al. (2011), Ravoet et al. (2015), Shi et al. (2016), Remnant et al. (2017), Roberts, Anderson & Durr (2017). A majority of accessions derived from Australia, but diversity in that country appears to be representative of the whole (Roberts, Anderson & Durr, 2017 and this study). The RDRP reference database used accessions generated by Webster et al. (2015), Daughenbaugh et al. (2015), Shi et al. (2016), Runckel et al. (2011), Roberts, Anderson & Durr (2017), Ravoet et al. (2015), and Li et al. (2017).

SRA accession information for *A. mellifera* (NCBI taxon 1758) was downloaded on April 1, 2018. SRA accessions were filtered to include runs on Illumina or ABI platforms with “transcriptomic” as the library source, and cDNA or polyA capture methods were excluded because RNA fragmentation provides more even recovery of RNA virus genomes (Wang, Gerstein & Snyder, 2009; Cornman, 2017). The number of accessions passing these filters was 363 (File S1), but 12 of these were excluded post hoc because the sequence data exceeded 15 GB in size. SRA accession information for *V. destructor* (NCBI taxon 109461) was downloaded on October 3, 2018, and filtered for platform only (due to the smaller number of accessions available), from which 57 accessions were identified (File S1).

For the selected accessions, up to the first 50 million reads or read pairs (minimum read length 50 bases) were searched for homology to LSV using Diamond v. 0.9.8 (Buchfink, Xie & Huson, 2014). The reference database (File S2) included RDRP sequences from multiple LSV lineages as well as related viruses identified by Shi et al. (2016) and the more distantly related chronic bee paralysis virus. The non-LSV sequences were included to help limit false-positive matches, as downloading, extracting, and assembling SRA accessions incurs a nontrivial computational cost. For *A. mellifera*, 31 of 351 accessions had match scores of at least 50 to an LSV accession, whereas 3 of 57 *V. destructor* accessions had equivalent matches. Accessions with matches were then downloaded in their entirety and assembled with Spades v. 3.11.0 (Bankevich et al., 2012), using a kmer of 27 and a contig coverage cutoff of 2×, and with the read-error correction module disabled.

### Generation of ORF alignments

Contigs from all assemblies were searched against the full-length ORFs downloaded from NCBI. Searches were again performed with Diamond and only matches with a bit score of 100 were retained initially, at which point multiple contigs typically matched each reference sequence per assembly but few were full length. As only near full-length ORFs were of interest for this study, contigs with matches less than 75% of the maximum length were excluded, as were contigs with nonhomologous sequence inserted (evident in alignments). As only one *V. destructor* SRA assembly produced contigs aligning to LSV ORFs, I limited the scope of the analysis to *A. mellifera* SRAs only. Importantly, only six BioProjects were represented by the 57 *V. destructor* SRA accessions, of which 40 derived from a single BioProject. I conclude that the available data are too limited to generalize as to the prevalence of LSV sequence reads in this species.

Exploratory alignments were constructed with ClustalW (Thompson, Higgins & Gibson, 1994) and edited with BioEdit (Hall, 1999) in order to identify and remove sequences that failed to align well across the length of ORFs. After this trimming, final full-length alignments were constructed with MUSCLE (Edgar, 2004) within MEGA7 (Kumar, Stecher & Tamura, 2016). Final alignments are provided in File S3. For phylogenetic analysis, concatenated alignments were made of ORF1 and RDRP, and of the capsid and ORF4, using BioEdit. The duplicated region of ORF1 and RDRP was included twice in that alignment, one copy in each of the two coding frames (this approach was preferred over deletion of the overlap region). Separate alignments of the 5′ and 3′ ORFs were needed to allow ORFs from partial genomes to be included in the phylogenetic analysis, with the goal of binning sequences into major clades comparable to the result of Bigot et al. (2017). The trees were created with MEGA7 using neighbor joining and amino-acid derived genetic distances based on the JTT exchange matrix. Rate variation was modeled using a gamma distribution with the shape parameter set to 0.5. This phylogenetic approach was used because it could be uniformly and flexibly applied to alignments of different lengths and ORF content, rather than attempting to optimize the evolutionary model in each case based on goodness of fit criterion. The latter would be problematic because the information available for model tests would be highly variable because of the strongly different lengths of alignments, and be further complicated by overlap regions in which two coding frames are present.

### Read-mapping to estimate relative abundance

Each SRA accession with matches to LSV was mapped to the two concatenated alignments (gaps and duplicate sequence removed). They were also mapped to all ORFs individually (not concatenated) to generate ORF-specific transcript levels irrespective of phylogenetic clade. Mapping was performed with Bowtie2 (Langmead & Salzberg, 2012) using the “sensitive” parameter settings and in “local” mapping mode (which allows reads extending beyond the edges of the reference sequences to be counted), with the “score-min” parameters set to “G,80,8.” Mappings were filtered on a Phred-scaled quality of 20 and tabulated with the idxstats command of samtools (Li et al., 2009). Counts were expressed as fragments per kilobase of references sequence per million mapped reads.

### Statistical analysis

Bootstrap support for phylogenetic trees was calculated for 1,000 resampled data sets. Relative codon usage (RCU) was calculated using the method of Stothard (2000) after concatenating all input sequences. Coding sequences for honey bee (official gene set v. 3.3; Elsik et al., 2014) and Varroa mite (https://i5k.nal.usda.gov/varroa-destructor) were downloaded from the i5K workspace (Poelchau et al., 2014). To ensure only coding portions of Varroa transcripts were analyzed, methionine-initiated ORFs of 100 codons or longer were extracted with the getorf program of the EMBOSS package (Rice, Longden & Bleasby, 2000); these were randomly downsampled fivefold to be comparable to the number of *A. mellifera* input sequences (13,567 and 15,314, respectively). To generate comparison codon usage data sets for which no ecologically relevant correlations are expected, coding sequences for *Drosophila melanogaster* and *Homo sapiens* were downloaded from BioMart (Smedley et al., 2009) on December 16, 2018. Methionine-initiated ORFs were then extracted and clustered at 95% identity with CD-HIT-EST (Fu et al., 2012), and then downsampled 10-fold.

Sliding windows of mean pairwise amino-acid divergence were calculated using a perl script to parse subsets of each ORF alignment and submit them to MEGA7. Recombination events between LSV1 and LSV2 were assessed with geneconv v. 1.81a (Sawyer, 1989), requiring a minimum tract length of 100 bases and a threshold *P*-value of 0.05, corrected for multiple comparisons. Relative rates of nonsynonymous substitution (the parameter ω) were estimated for each ORF alignment with PAML 4.9 (Yang, 2007) using eight gamma-distributed rate categories under Model 7 and Model 8. The latter model differs from the former in that the highest ω category is constrained to be greater than one, and the relative likelihoods of each given an input tree provides one possible test of positive selection. Protein structure predictions were performed on the PredictProtein server (Rost, Yachdav & Liu, 2004) using the method of Rost & Sander (1993).

## Results

### Distribution of LSV clades in SRA accessions

**Figure 1:**
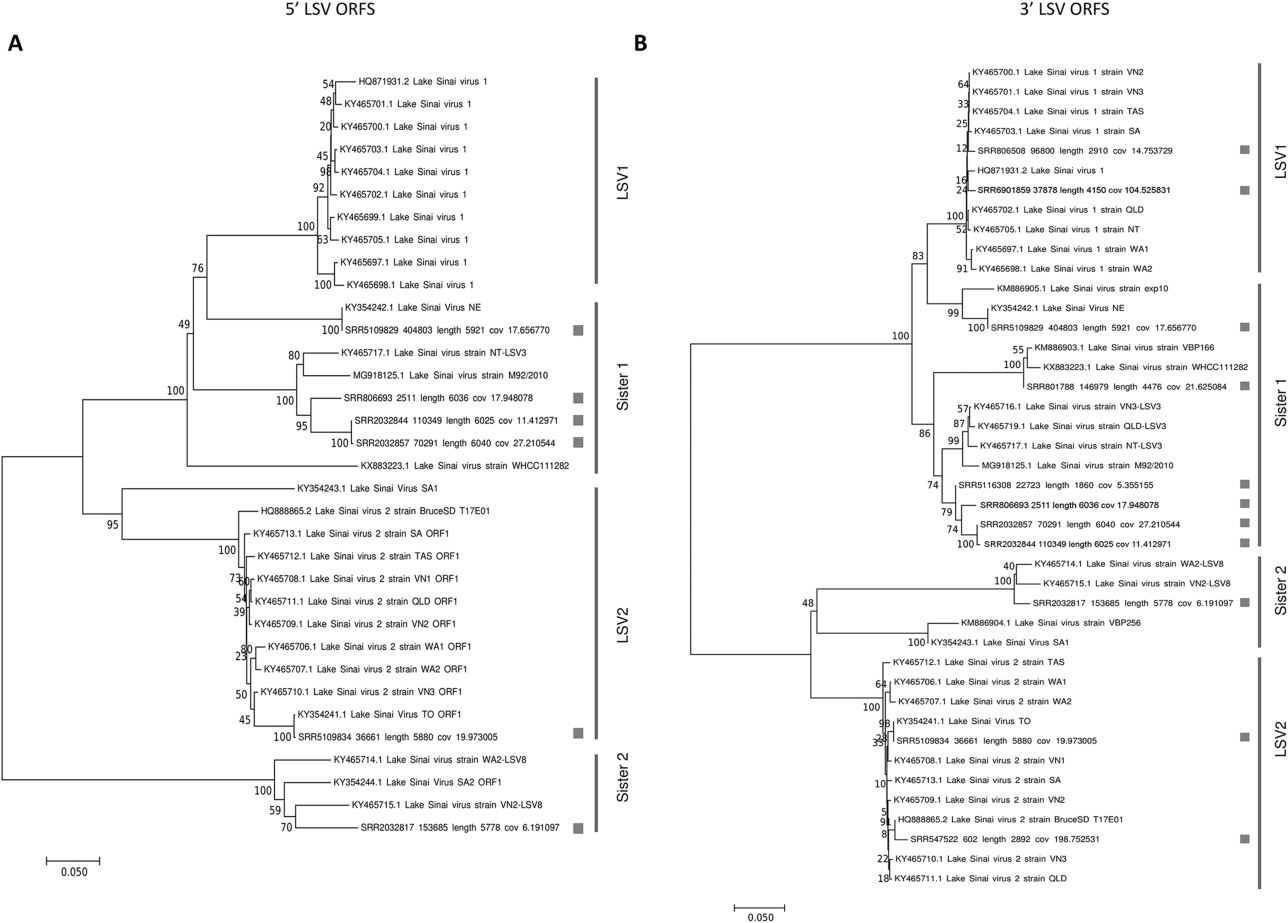
Neighbor-joining phylogenetic trees of LSV and operational binning into four clades based on existing LSV1 and LSV2 nomenclature. Trees were computed from predicted amino-acid sequences and using the JTT distance matrix. A gamma distribution of rate heterogeneity was assumed with a parameter value of 0.5. Bootstrap values are based on 1,000 resampled replicates. (A) Tree derived from 5′ ORFs (ORF1 and RDRP). (B) Tree derived from 3′ ORFs (capsid and ORF4).

Phylogenies of the concatenated ORF1 and RDRP coding sequence (5′ ORFs) and the concatenated capsid and ORF4 sequence (3′ ORFs) were concordant with each other (Fig. 1). Trees for each ORF separately are also provided as more sequences can be included in these shorter alignments (Fig. S1). The trees recovered well-supported clusters of sequences with short branch lengths that correspond to the LSV1 and LSV2 lineages (based on the annotations provided by their submitters). Although diagnostic sequence characteristics for these taxa have not been described, I operationally designate these clusters LSV1 and LSV2 as shown in Fig. 1 for the present analyses. Sequences sister to these clusters I have denoted “Sister 1” and “Sister 2,” respectively. This four-clade topology accords with previous results based on RDRP amino-acid sequence (Roberts, Anderson & Durr, 2017) and ORF1/RDRP nucleotide sequence (Bigot et al., 2017), but the current results verify that this phylogenetic pattern is consistent across the genome. This is important because ORF4 is a derived genomic feature that might bear a different evolutionary history, and because the ORF1/RDRP region includes a hypervariable region that may be involved in recombination events (see below), potentially complicating phylogenetic inference.

**Figure 2:**
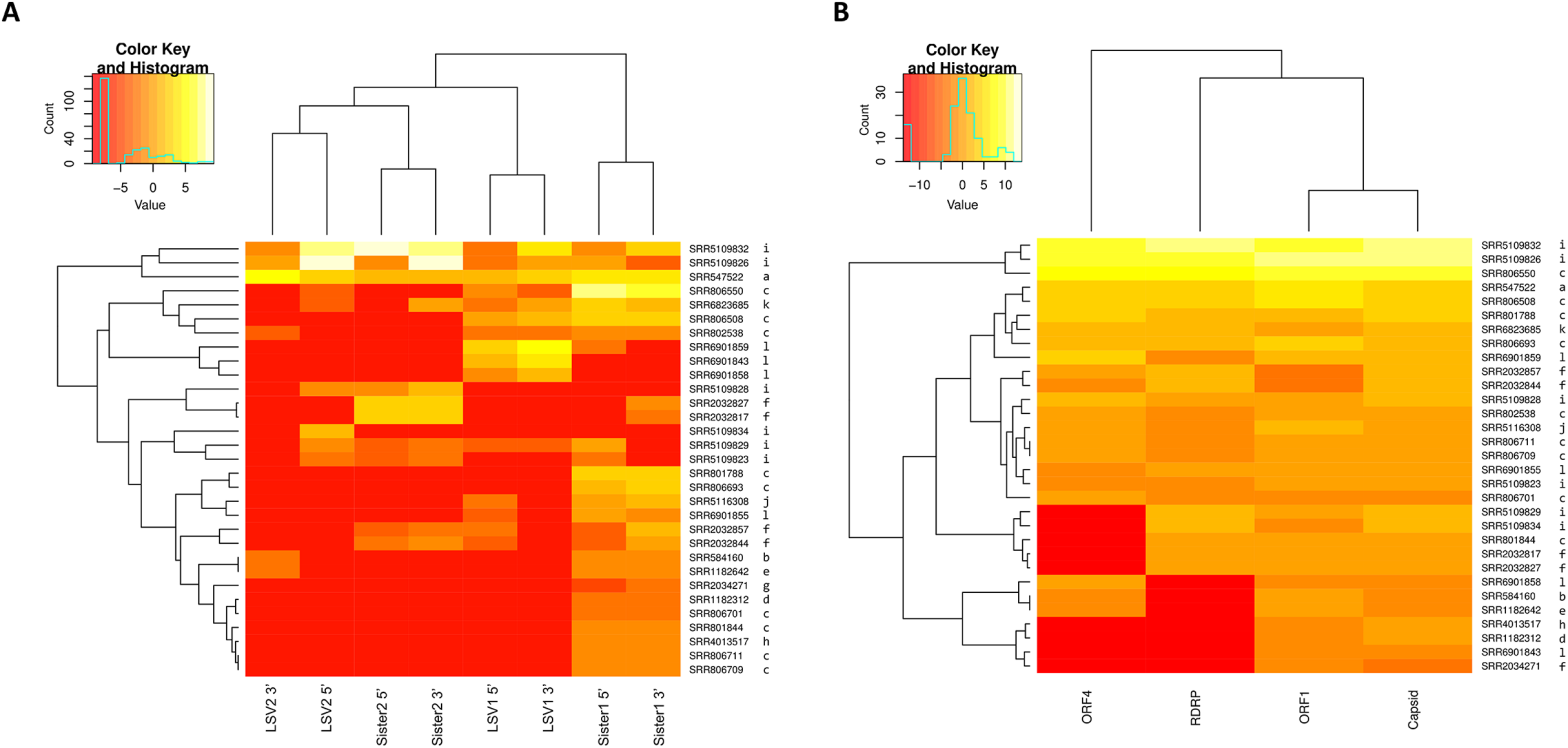
Heat maps of LSV read abundance in SRA accessions. SRA accession numbers are indicated on the right of the map and ordered by hierarchical clustering of Euclidean distance. The letter codes to the right of each accession correspond to separate BioProjects as follows: a, PRJNA172020; b, PRJNA175445; c, PRJNA194157; d, PRJNA238833; e, PRJNA240064; f, PRJNA277772; g, PRJNA284414; h, PRJNA338112; i, PRJNA357165; j, PRJNA357705; k, PRJNA437728; l, PRJNA445764. Input values were fragments per kilobase per million mapped reads (FPKM). A pseudocount of 0.001 was added to each value to eliminate zero values and thereby smooth the color scale. The darkest color should therefore be interpreted as “not detected”. (A) Counts for 5′ and 3′ genomic regions of each clade, which were binned separately according to the designations in Fig. 1. (B) Counts for each ORF summed across all clades.

The relative abundance of SRA sequence reads mapped to reference sequences binned as shown in Fig. 1 suggests that “Sister 1” lineages are more prevalent than other groups (Fig. 2A; *N* = 31 accessions). The 5′ and 3′ genomic regions of each clade clustered together in the heatmap and their representation appears approximately even, suggesting that any technical bias in their relative rate of recovery is likely small (Fig. 2A). This observation increases confidence that the variation in ORF transcript abundance evident in Fig. 2B reflects genuine variation in expression. For example, ORF4 expression appears divergent from and more bimodal than capsid transcript expression, despite their physical linkage. This contrasts with Runckel et al. (2011), who found no evidence of subgenomic transcripts by Northern blot.

### Tests of recombination between LSV1 and LSV2

For the RDRP, capsid, and ORF4 alignments, no intra-ORF recombination events of 100 bases or more were detected between LSV1 and LSV2. Evidence was found for ORF1 between those groups (File S4), however both significant recombination events had estimated break points within a hypervariable region of the ORF1/RDRP overlap (described below). The nucleic acid test of (Sawyer, 1989) is based on silent site distributions, which are not correctly specified when more than one frame is coding, and further assumes that variation is independent of position within an alignment, which empirical evidence suggests is not valid either, as will be shown below. Thus, the recombination tests by themselves provide only tentative evidence of intergroup recombination in the ORF1/RDRP overlap region. Note that analysis of the combined ORF1/RDRP region and all concatenated ORFs did not alter the result, indicating that it is robust to changes in the length and overall variability of the sequences considered (File S4).

Curiously, contigs removed from ORF alignments due to the presence of nonhomologous sequence (see Materials and Methods) provide some corroborative evidence of genomic breakpoints and exchange occurring in this region (Fig. S2). Multiple assemblies recovered contigs that were structurally variable 5′ of the ORF1/RDRP overlap, including instances in which RDRP sequence fragments were integrated in-frame within ORF1. While it is not unexpected that viral metagenomic assemblies would recover nonhomologous mosaic sequences, either due to assembly error or due to chance mosaics generated during viral replication, the three examples shown in Fig. S2 were all recovered in multiple accessions, with identical break points and coding sequence and without frameshift. That each occurrence is a unique event is evident from their distinct nucleotide compositions. The biological significance of these chance observations and the frequency of their occurrence remain to be determined, but collectively they provide evidence of a hotspot of genomic breakage consistent with both homologous and nonhomologous exchange.

### The ORF1/RDRP overlap encodes a hypervariable protein segment

**Figure 3:**
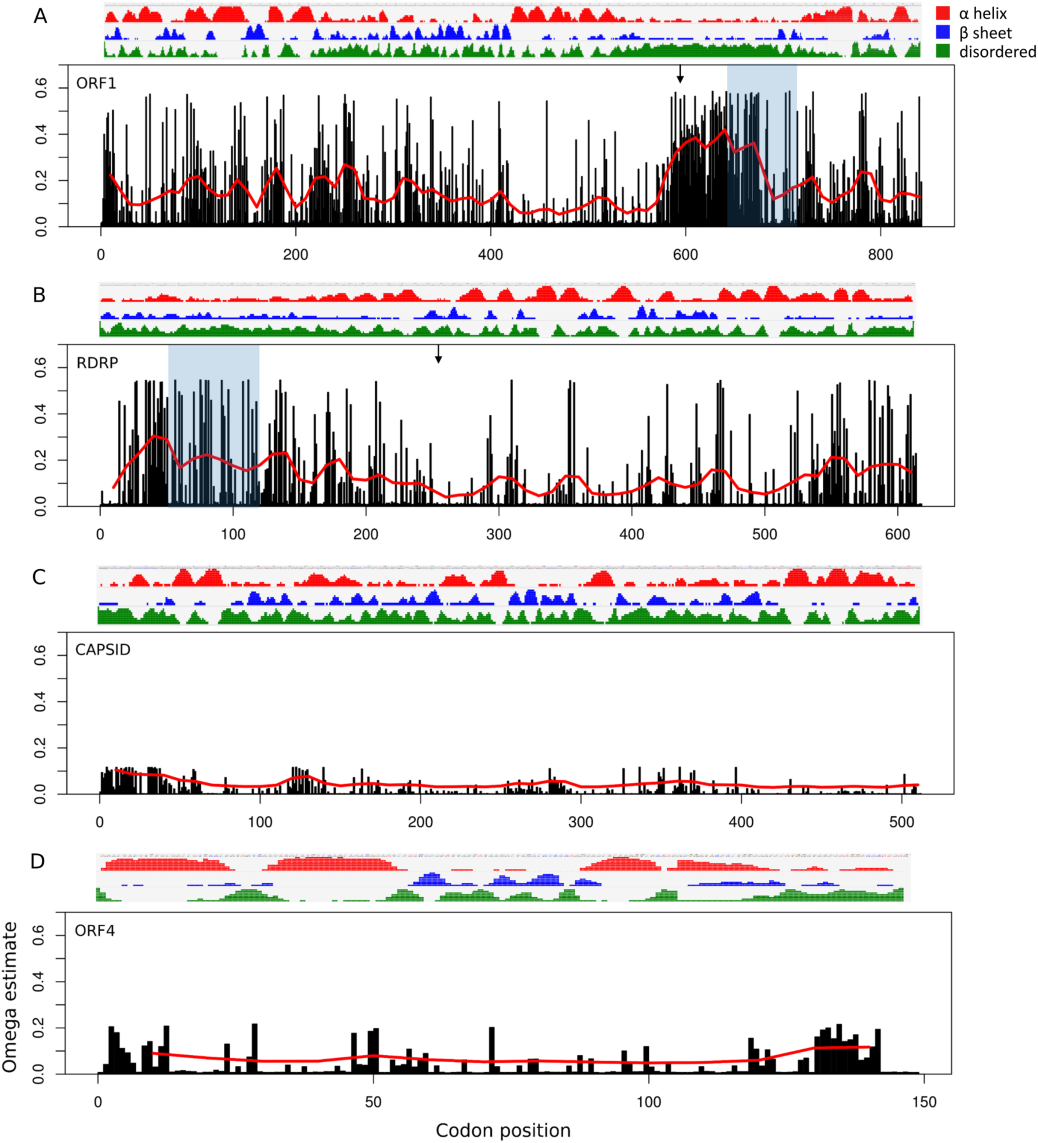
Relative rate of substitutions (ω) by codon along each ORF. Eight categories of ω were estimated with PAML under Model 7, and site-specific ω are the probability-weighted average ω across all categories. Note the scale of the vertical axis is the same in all four panels. (A) ORF1, (B) RNA-dependent RNA polymerase (RDRP), (C) Capsid, and (D) ORF4.

**Figure 4:**
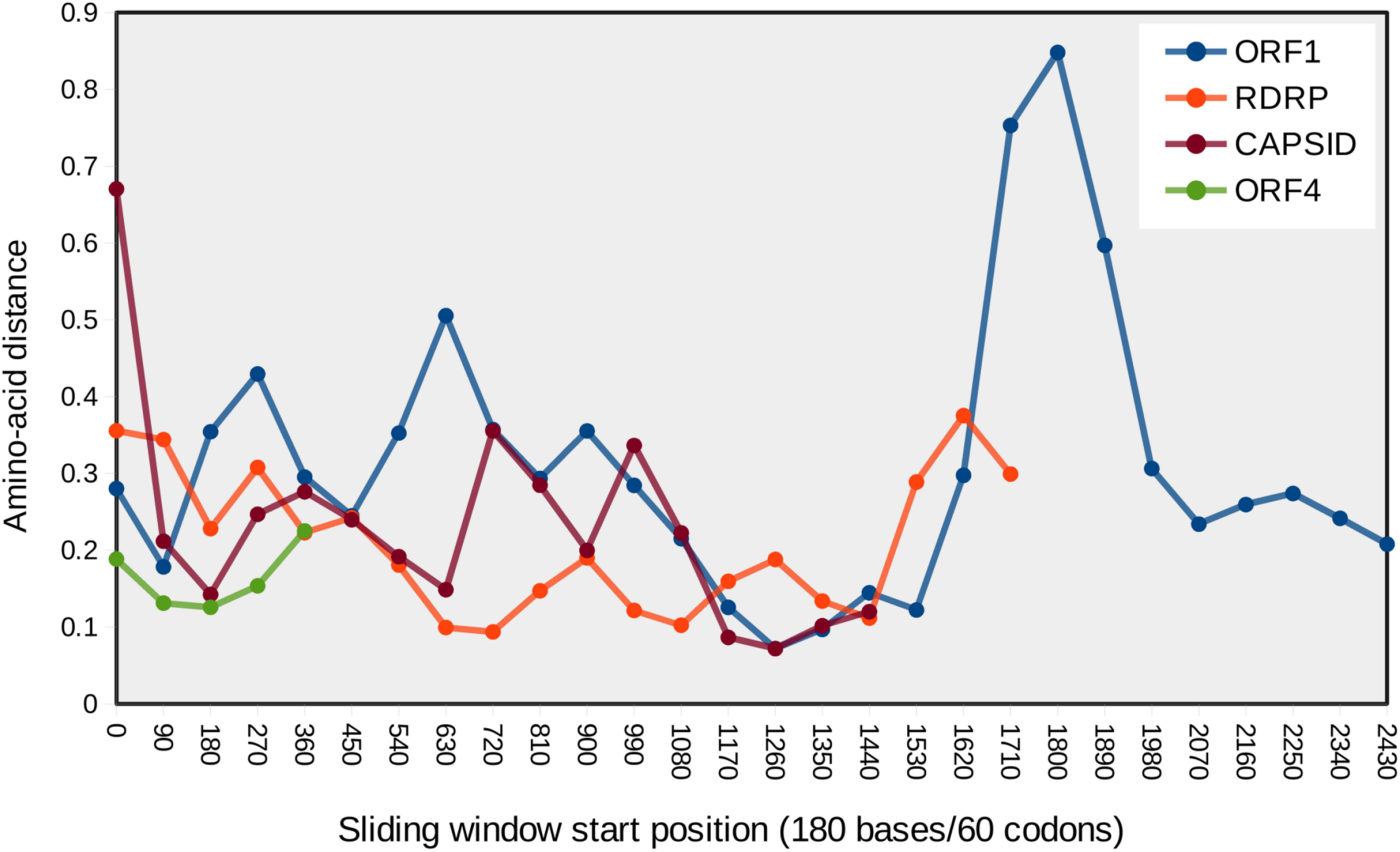
Mean pairwise protein divergence among ORF sequences in sliding windows of 60 codons (180 bases), using the JTT distance metric. A peak of amino acid divergence is evident for ORF1 around nucleotide position 1800.

Site-specific evolutionary rates (ω distribution under PAML Model 7) estimated for each ORF separately revealed that the 5′ ORFs have proportionally more codons with relaxed constraint than do the 3′ ORFs (Fig. 3). The average ω across all sites was 0.150 and 0.119 for ORF1 and RDRP, respectively, whereas it was 0.024 and 0.049 for the capsid and ORF4, respectively (Table 1). The mean ω in sliding windows of 20 codons demonstrates that a region of ORF1 is evolving more rapidly (red line in Fig. 3). This empirical pattern may have impacted the recombination tests, as alluded to previously, because local polymorphisms should exceed the genome-level average. Furthermore, the amino-acid changes in this region are not conservative, as indicated by sliding window analysis of protein divergence (Fig. 4) using the JTT distance metric, which weights substitutions by evolutionary exchangeability (Jones, Taylor & Thornton, 1992). Nonetheless, likelihoods estimated for an evolutionary model with some sites under positive selection (PAML Model 8) were not significantly greater than for a model lacking such sites (PAML Model 7) for any ORF (Table 1). Protein secondary structure prediction indicated that the hypervariable region is likely disordered in both ORF1 and RDRP (Fig. 3).

**Table 1:** Estimates of ω and tree likelihoods for each ORF under the M7 and M8 models in PAML.

Region	Average ω of M7 model	ln(L) of M7 model	Test statistic for comparison with M8 model*
ORF1	0.150	−24,414.5037	0.664
RDRP	0.119	−19,717.6734	0.569
Capsid	0.024	−16,610.3709	−0.782
ORF4	0.049	−3,920.7958	−0.298

**Note:**

* The test statistic is twice the difference in ln(likelihood) and the critical value is approximated by χ^2^ for α = 0.05 and d*f* = 2, or 5.991 (Yang, 2007).

### LSV codon usage is uncorrelated with honey bee usage

**Figure 5:**
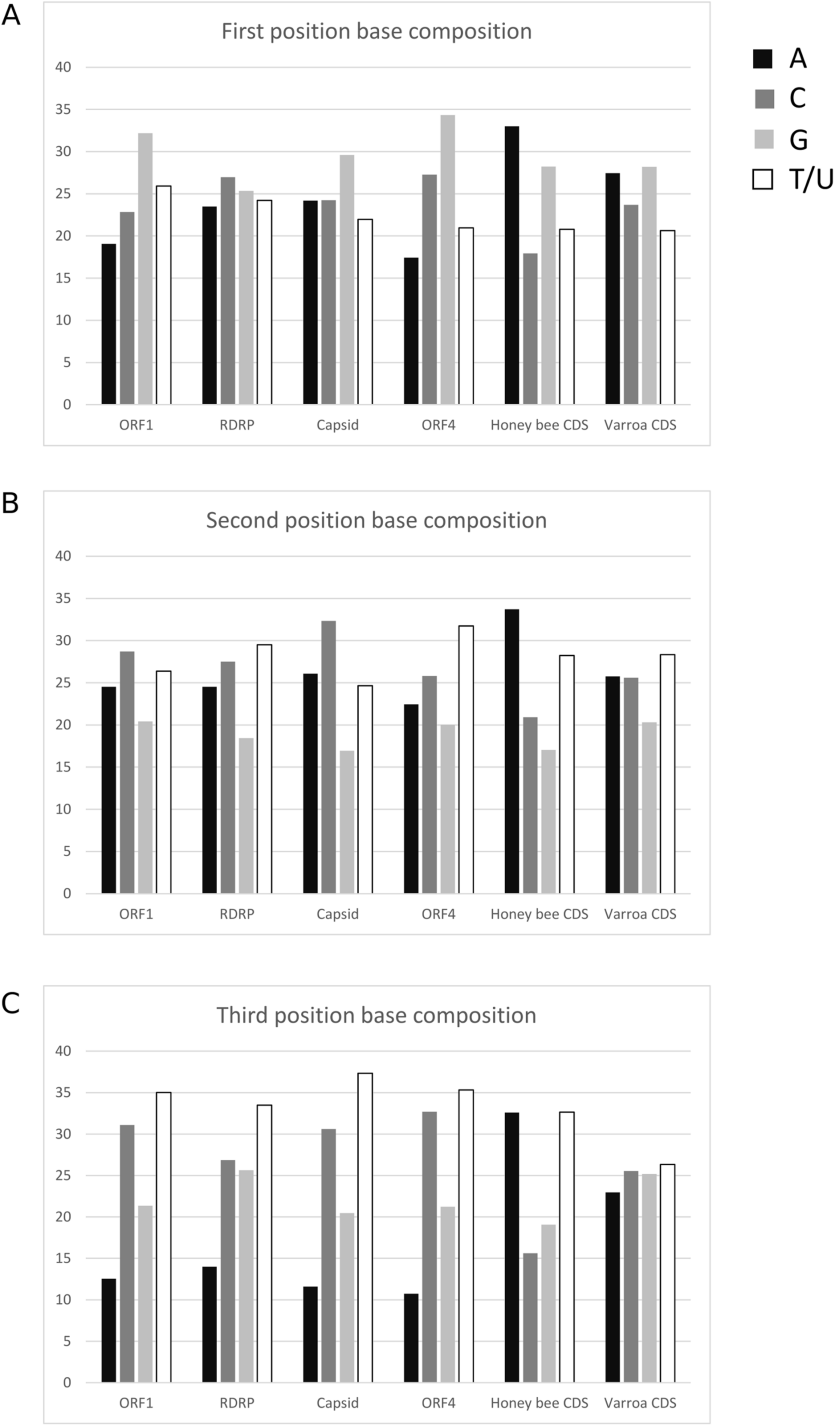
Nucleotide composition at codons positions of each LSV ORF and averaged across honey bee and Varroa mite coding sequences. (A) First position of codons, (B) second position of codons, and (C) third position of codons.

The average base composition at each codon position is similar among LSV ORFs but differs from both *A. mellifera* and *V. destructor* coding sequence (Fig. 5). This lack of convergence by LSV is in striking contrast to that exhibited by common honey bee Iflaviruses and Dicistroviruses (cf. Fig. 1 of Chantawannakul & Cutler (2008)). Further, RCU of LSV is uncorrelated with *A. mellifera* RCU but is correlated with *V. destructor* RCU. The Pearson correlation coefficient for LSV and *A. mellifera* was −0.0333 (95% CI [−0.287–0.225], two-sided *P* = 0.804) and 0.404 (95% CI [0.165–0.598], two-sided *P* = 0.0015) for LSV and *V. destructor*. The lack of a correlation with *A. mellifera* codon usage is again in contrast to Iflaviruses and Dicistroviruses that infect honey bee (cf. Fig. 3 of Chantawannakul & Cutler (2008)). The correlation with *V. destructor* is intriguing considering that Varroa is itself only weakly biased at third codon positions and in RCU. However, *V. destructor* and LSV had similar third position GC content overall in the tested data sets (48% and 49%, respectively) and differed substantially from the third position GC content of the *A. mellifera* data set (35%). As base composition strongly impacts codon usage generally (Novembre, 2002), the different strengths of correlation between LSV and the two arthropod species may be attributable to this factor. For comparison, LSV RCU was uncorrelated with that of two “control” species for which no ecologically relevant correlation was expected: *D. melanogaster* (0.043, 95% CI: [−0.215–0.296], two-sided *P* = 0.744) and *H. sapiens* (−0.171, 95% CI [−0.409–0.089], two-sided *P* = 0.196).

**Figure 6:**
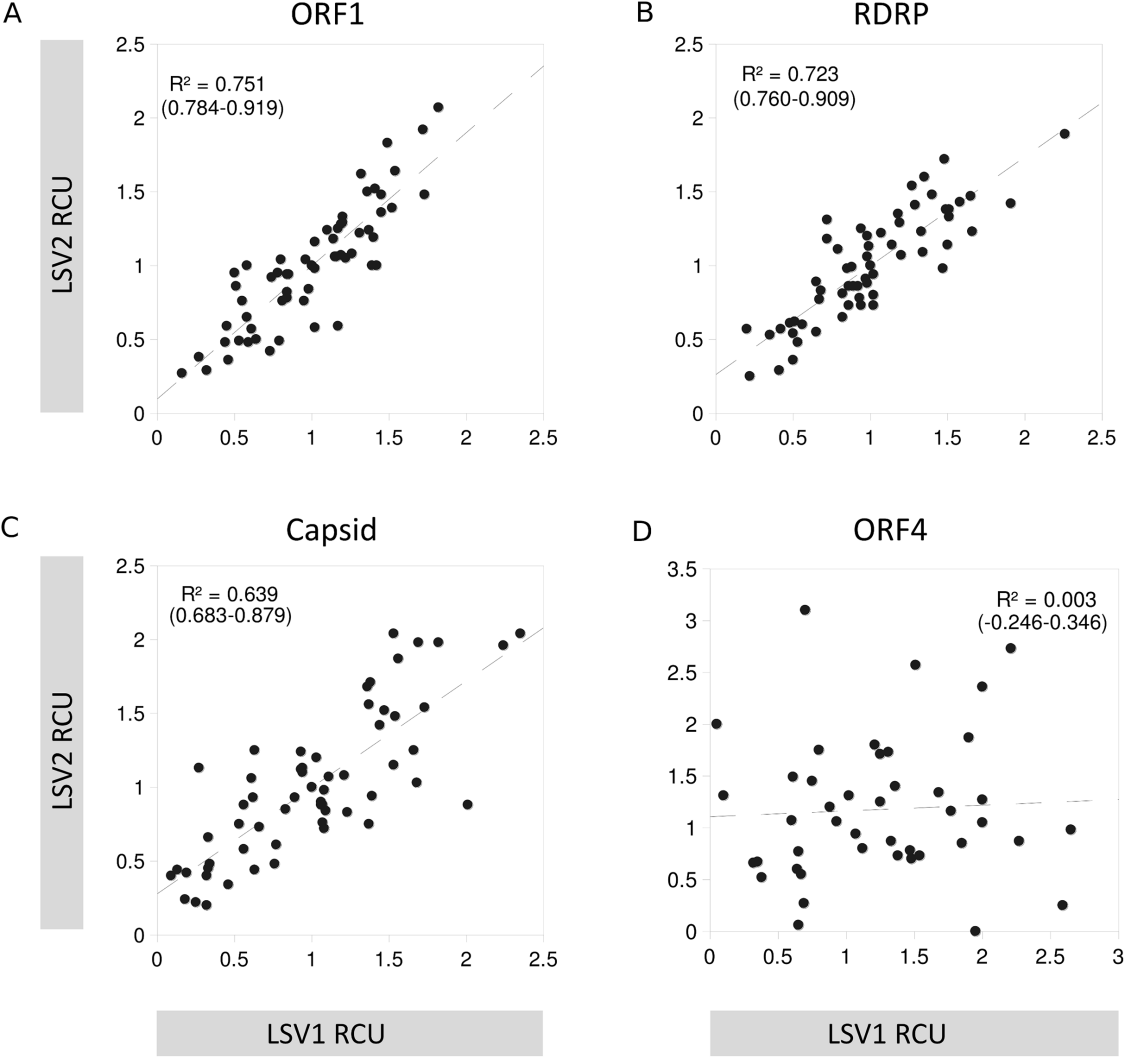
Codon usage similarity between LSV1 and LSV2 for each LSV ORF. Scatterplot points represent the RCU value of individual codons in each of the two groups compared, and the dashed line represents the slope of the relationship. Also shown are the *R*^2^ of each correlation and the 95% confidence interval for R. Codons for methionine and tryptophan are excluded because RCU is necessarily one (no alternative codons occur). (A) ORF1, (B) RNA-dependent RNA polymerase (RDRP), (C) Capsid, and (D) ORF4.

For three of the four ORFs, the LSV1 and LSV2 lineages have similar RCU, but RCU is uncorrelated between these groups for ORF4 (Fig. 6), implying substantial change in codon usage since the divergence of these viral types. As GC content of ORFs are very similar between lineages, base composition can be discounted as the underlying driver of this difference. Note that because ORF4 is substantially shorter than other LSV ORFs, some codons occur very infrequently among the aligned sequences, which can inflate Pearson correlations due to frequent pairing of values near zero. I therefore excluded codons from the ORF4 calculation that had a mean number of occurrences smaller than the smallest value for the other three ORFs (1.4).

## Discussion

### LSV diversity

This study used public sequence data to investigate the molecular evolution of LSV and the relative abundance of ORFs and clades within SRA accessions. The use of SRA data helped ensure LSV diversity was as fully represented as possible. The phylogenies produced here with additional ORF sequence were consistent with the topologies reconstructed by Bigot et al. (2017) and Roberts, Anderson & Durr (2017) based on narrower genomic regions, again identifying sequence clusters corresponding to the LSV1 and LSV2 designations as well as longer-branch sister groups of each. While isolates with LSV1 and LSV2 designations are clearly distinct sequence clusters, they are embedded within a diverse set of sister lineages for which there seems little basis as yet for further differentiation or nomenclature. Indeed, this view is consistent with current naming conventions within the NCBI taxonomy, which identifies LSV1 and LSV2 but places other isolates named in the literature under “unclassified Sinaivirus” (National Center for Biotechnology Information, 2018). Note that a common phylogenetic method was used for all trees, but with rate categories estimated individually for each tree. Optimal models for phylogenetic inference were not fitted to each ORF alignment because they varied in length, composition, and availability of outgroups. Subsequent work will surely improve these evolutionary reconstructions, the purpose here being to bin partial sequences in a manner consistent with what is known about the phylogenetic pattern of the viruses as a whole (Bigot et al., 2017), while also recognizing that the placement of individual sequences may be ambiguous or subject to revision with additional data.

Of the 17 sequences reconstructed from SRA accessions that were placed in Fig. 1, 12 fell within lineages sister to LSV1 and LSV2, perhaps suggesting an ascertainment bias against their detection by non-metagenomic survey methods. Phylogenies for individual ORFs, which are based on more SRA accessions, also support this interpretation, with the possible exception of the RDRP tree (Fig. S1). For the other three ORFs, 24 of 35 sequences were sister to LSV1 or LSV2, whereas for RDRP, a large cluster of sequences derived from SRA accessions that were sister to LSV1, but were only slightly diverged such that I classified them operationally as LSV1. The apparent under-representation of LSV sister lineages in public databases is further suggested by read-mapping to binned reference sequences, which detected lineages sister to LSV1 most frequently. Time of sampling or some other ecological factor may contribute to ascertainment biases. For example, LSV1 and LSV2 were found to have very different seasonal distributions in a temporal analysis by Runckel et al. (2011), with LSV1 common and LSV2 rare during summer months. However, a much larger analysis found LSV2 abundance in summer to be typical of other common pathogens (Traynor et al., 2016). Furthermore, longitudinal analysis of almond pollination hives (Cavigli et al., 2016, Glenny et al., 2017) found differences in LSV1 and LSV2 distributions that were attributable to apiary operator but not to season. Thus, it remains unclear what factors structure LSV1 and LSV2 distributions and how they might bias metagenomic detection. Read mappings were in fact consistent with frequent co-infection, which has been reported previously (Ravoet et al., 2015), although whether a given SRA accession derives from an individual or pools of bees is not necessarily known, as sample metadata are not uniformly provided.

Recombination did not appear to contribute significantly to the origin of lineages sister to LSV1 and LSV2. Recombination was evaluated only between LSV1 and LSV2 in part for feasibility, because the uniformity of those clusters should make recombinants more evident. It was also partly for relevance, because it is the intermediate phylogenetic positions and long-branches of sister lineages that suggest recombinant origins. Given these considerations, small recombination tracts between more closely related viruses were not considered, as they would be more challenging to differentiate robustly from other processes and would contribute relatively little to the overall phylogenetic diversity observed. Patterns of co-occurrence suggest that there is reasonable opportunity for recombination between divergent lineages, and the observation in this study of recurring mosaicism and structural variation suggests hotspots of nonhomologous exchange may exist.

### Rates of amino-acid divergence among LSV ORFs

Use of an evolutionary model incorporating rate variation among sites, which is more reasonable than a single ω parameter fitted to all sites, further confirmed that the nonstructural proteins ORF1 and RDRP have evolved at a somewhat higher rate than the capsid and ORF4. While ORF4 is a derived sequence acquired since divergence from other known viral families and its biological functions remain unknown, the degree of evolutionary constraint is comparable to that of the structural capsid protein.

The significance of the hypervariable region within ORF1 is unclear, however, the very low probability of alpha and beta structures in the region suggests that it is predominantly disordered and may not be critical to protein tertiary structure. If recombination events are in fact frequent in this region, this process may contribute to the apparent mutability as well. Selection might also be occurring directly on the nucleotide sequence rather than amino-acid sequence, for which codon-based models would be uninformative. Nucleotide sequence variation in this region might affect genome conformation, transcription initiation, or some other protein-RNA interaction, for example.

### LSV codon usage is not covolved with honey bee hosts

Neither base composition by codon position nor RCU were similar between LSV and *A. mellifera*. This contrasts with the pattern evident in established honey bee pathogens such as deformed wing virus and Israeli acute paralysis virus. The lack of adaptation to honey bee hosts in this regard suggests that other hosts with different genomic characteristics have shaped LSV evolution, either currently or in the recent past. *V. destructor* is another potential host although the negative strand replication intermediate has not been detected (Daughenbaugh et al., 2015; Ravoet et al., 2015). While LSV RCU is significantly correlated with *V. destructor* RCU despite differences in third position base composition, the overall GC contents of the tested coding sequences were similar and differed from the more AT-rich honey-bee coding sequence. This compositional similarity may be sufficient to explain the greater correlation observed. No positive relation between LSV and mite prevalence has been seen in surveys (e.g., Traynor et al., 2016; Glenny et al., 2017) and LSV is both abundant and diverse where *V. destructor* has been historically absent (Remnant et al., 2017; Roberts, Anderson & Durr, 2017). It should also be noted that codon covolution is not universal in insect viruses (Chantawannakul & Cutler, 2008).

Other potential hosts of LSV have been identified, including ants (Bigot et al., 2017) and other bees (Ravoet et al., 2015, Parmentier et al., 2016), and replication intermediates have been detected in *Osmia cornuta* (Ravoet et al., 2015). Further metagenomic studies may shed light on the range of LSV hosts and explain why LSV differs from other honey bee viruses in this respect. This approach may also help explain why ORF4 RCU is not conserved among LSV1 and LSV2, in contrast to other ORFs. Although the small number of ORF4 codons promotes the stochastic divergence of this statistic, rare codons were excluded from the correlation to mitigate this effect. Unfortunately, LSV was detected in only three SRA accessions of *V. destructor*, two at very low levels, such that the data are insufficient at present to compare LSV isolates between these two hosts.

## Conclusion

Lake Sinai Virus is a persisting metagenomic mystery of honey bees, in that it appears to be highly abundant in both weak and healthy colonies (Cornman et al., 2012; Daughenbaugh et al., 2015; Glenny et al., 2017), is highly diverse relative to other honey bee RNA viruses, and superinfections at the level of the colony and the individual bee appear common. It remains unclear whether *A. mellifera* is the primary host of all LSV clades detected to date and how frequently replication occurs in other species. This study further clarified the relative distribution of LSV clades and identified patterns of molecular evolution that can guide future investigations of function and adaptation.

## Supplemental Information

10.7717/peerj.6305/supp-1 Supplemental Information 1 Run metadata for *Apis mellifera* and *Varroa destructor* SRA accessions. These accessions were selected after applying filters as described in the Materials and Methods. Note that SRA information was downloaded on different dates for the two species, and thus have slightly different headers and metadata formats. A few metadata columns not relevant to the study have been omitted.

10.7717/peerj.6305/supp-2 Supplemental Information 2 RDRP protein sequences used as the reference database for detection of Lake Sinai Virus in SRA accessions.

10.7717/peerj.6305/supp-3 Supplemental Information 3 ORF sequences used in analyses. Six sets of sequences are contained in this fasta file. Each set begins with a prefix: ORF1, RDRP, Capsid, ORF4, 3Prime-merged, or 5Prime-merged. Sequences are aligned within each set. Sequences derived from SRA accessions begin with the accession number and the contig identifier generated by assembly with Spades (Bankevich et al., 2012).

10.7717/peerj.6305/supp-4 Supplemental Information 4 Output of the program geneconv v. 1.81a (Sawyer, 1989) for LSV ORF alignments. The column headers indicate the two sequence pairs for which a statistic was reported, the operational binning of that sequence following Fig. 1 and Fig. S1, the two *P*-values reported by geneconv that use alternative false-discovery corrections, start and end coordinates of the proprosed recombinant fragment, its length and polymorphic site number. Rows highlighted in green passed all three filters imposed: a length of 100 bases or more, both *P*-value calculations < 0.05, and both LSV1 and LSV2 represented among participating sequences. Rows that failed these criteria are denoted by orange highlighting in the column that failed this filtering.

10.7717/peerj.6305/supp-5 Supplemental Information 5 Amino-acid based phylogenies of individual LSV ORFs. Trees were computed from predicted amino-acid sequences and using the JTT distance matrix. A gamma distribution of rate heterogeneity was assumed with parameter 0.5. Bootstrap values are based on 1,000 resampled replicates.

10.7717/peerj.6305/supp-6 Supplemental Information 6 Examples of mosaicism with assembled contigs.

## Additional Information and Declarations

### Data Availability

The following information was supplied regarding data availability: All primary data used are public accessions already in GenBank, and listed in the manuscript or its supplements. The additional sequences generated were metagenomic contigs. NCBI per policy does not house metagenomic assemblies of this nature. The relevant contigs are included as a supplement. The data used in this study are also available as a U.S. Geological Survey data release (Cornman, 2018).
