# Supplementary material for: Relative abundance and molecular evolution of Lake Sinai Virus (Sinaivirus) clades
Source: PeerJ. 2019 Mar 21;7:e6305. doi: 10.7717/peerj.6305 (PMC6431542; doi:10.7717/peerj.6305)
Supplement: Supplemental Information 5 — Trees were computed from predicted amino-acid sequences and using the JTT distance matrix. A gamma distribution of rate heterogeneity was assumed with parameter 0.5. Bootstrap values are based on 1,000 resampled replicates. [file peerj-07-6305-s005.pdf]

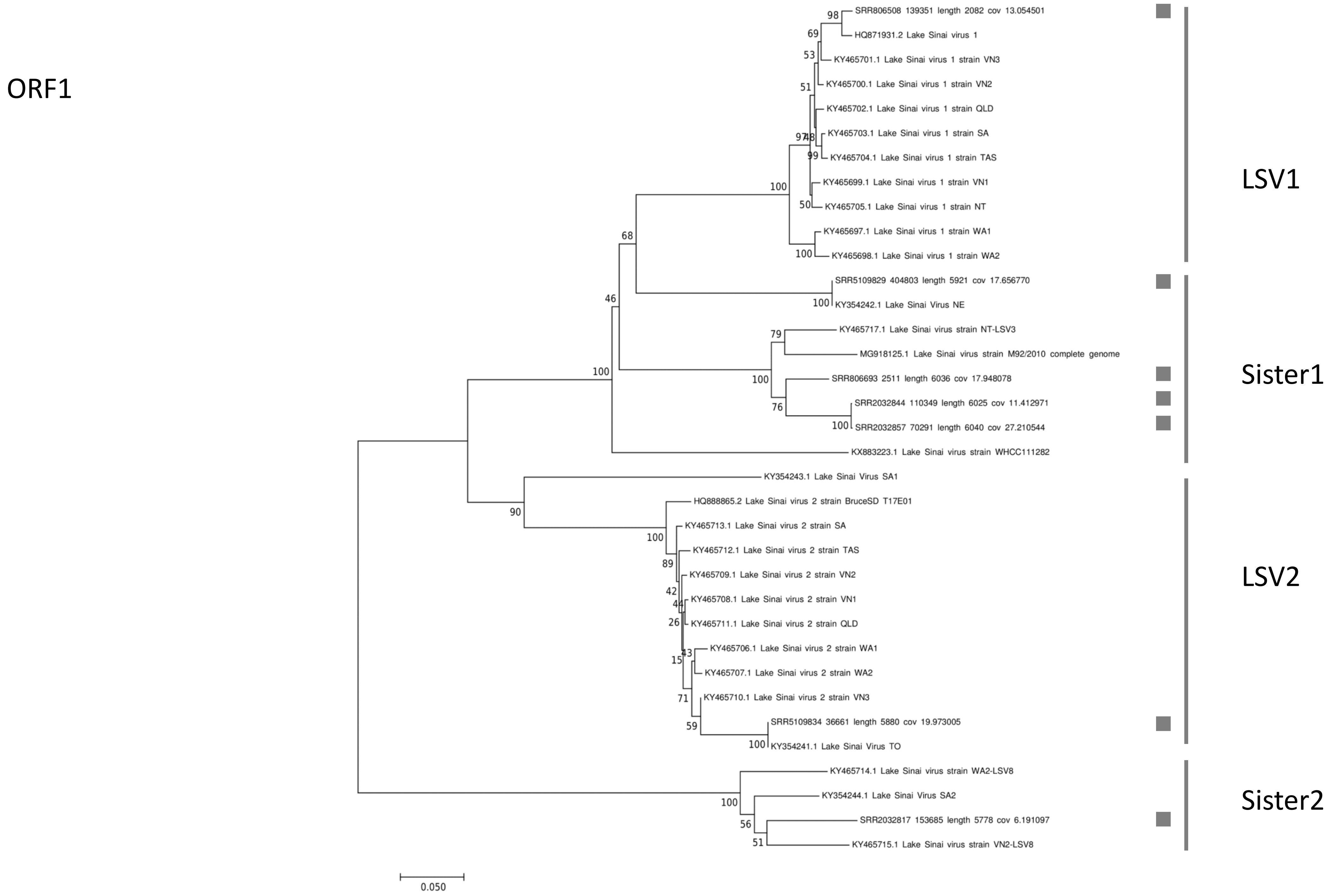

ORF1
SRR806508 139351 length 2082 cov 13.054501
HQ871931.2 Lake Sinai virus 1
KY465701.1 Lake Sinai virus 1 strain VN3
KY465700.1 Lake Sinai virus 1 strain VN2
KY465702.1 Lake Sinai virus 1 strain QLD
KY465703.1 Lake Sinai virus 1 strain SA
KY465704.1 Lake Sinai virus 1 strain TAS
KY465699.1 Lake Sinai virus 1 strain VN1
KY465705.1 Lake Sinai virus 1 strain NT
KY465697.1 Lake Sinai virus 1 strain WA1
KY465698.1 Lake Sinai virus 1 strain WA2
SRR5109829 404803 length 5921 cov 17.656770
KY354242.1 Lake Sinai Virus NE
KY465717.1 Lake Sinai virus strain NT-LSV3
MG918125.1 Lake Sinai virus strain M92/2010 complete genome
SRR806693 2511 length 6036 cov 17.948078
SRR2032844 110349 length 6025 cov 11.412971
SRR2032857 70291 length 6040 cov 27.210544
KX883223.1 Lake Sinai virus strain WHCC111282
KY354243.1 Lake Sinai Virus SA1
HQ888865.2 Lake Sinai virus 2 strain BruceSD T17E01
KY465713.1 Lake Sinai virus 2 strain SA
KY465712.1 Lake Sinai virus 2 strain TAS
KY465709.1 Lake Sinai virus 2 strain VN2
KY465708.1 Lake Sinai virus 2 strain VN1
KY465711.1 Lake Sinai virus 2 strain QLD
KY465706.1 Lake Sinai virus 2 strain WA1
KY465707.1 Lake Sinai virus 2 strain WA2
KY465710.1 Lake Sinai virus 2 strain VN3
SRR5109834 36661 length 5880 cov 19.973005
KY354241.1 Lake Sinai Virus TO
KY465714.1 Lake Sinai virus strain WA2-LSV8
KY354244.1 Lake Sinai Virus SA2
SRR2032817 153685 length 5778 cov 6.191097
KY465715.1 Lake Sinai virus strain VN2-LSV8
LSV1
Sister1
LSV2
Sister2
0.050

RDRP

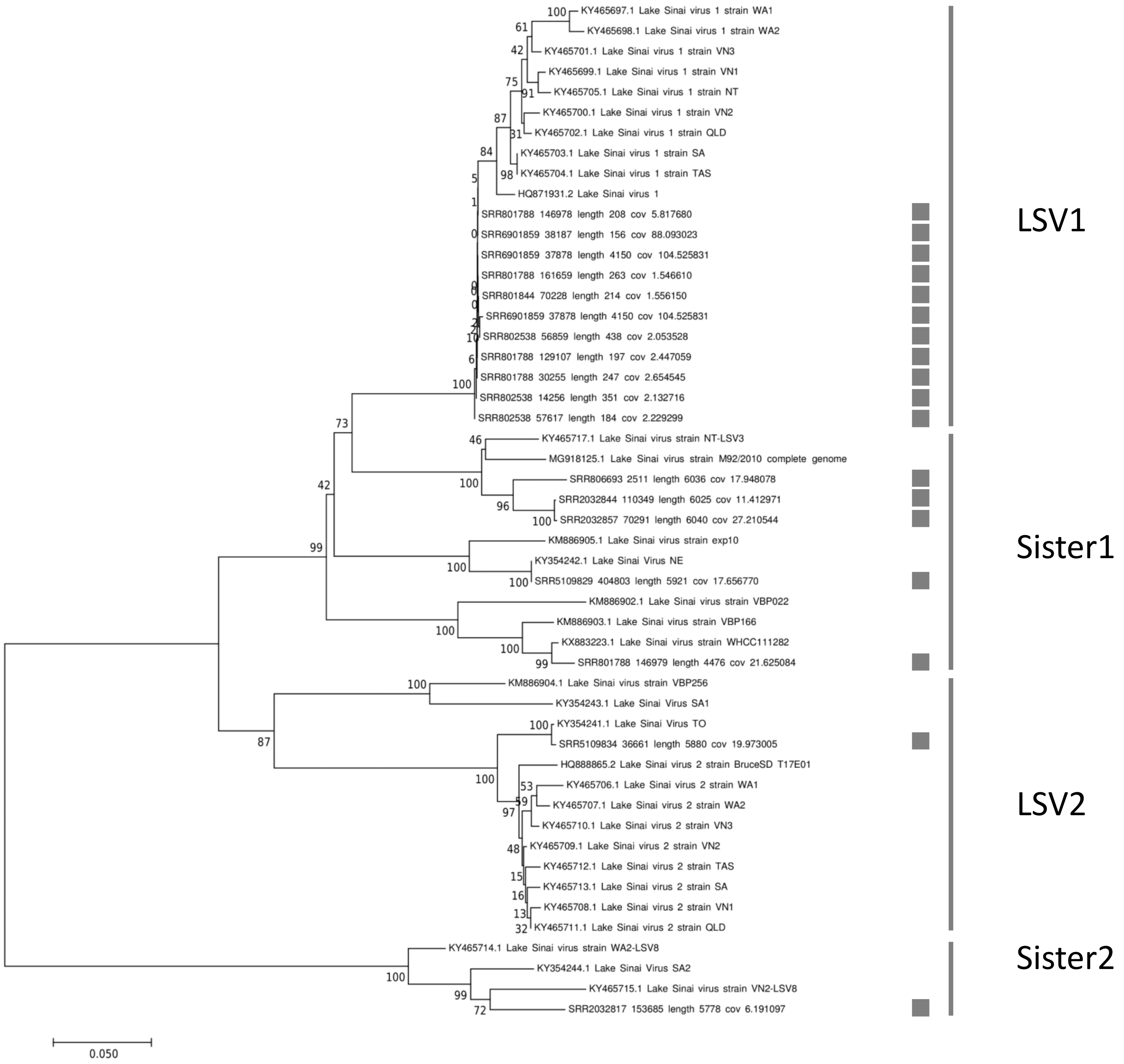
KY465697.1 Lake Sinai virus 1 strain WA1
KY465698.1 Lake Sinai virus 1 strain WA2
KY465701.1 Lake Sinai virus 1 strain VN3
KY465699.1 Lake Sinai virus 1 strain VN1
KY465705.1 Lake Sinai virus 1 strain NT
KY465700.1 Lake Sinai virus 1 strain VN2
KY465702.1 Lake Sinai virus 1 strain QLD
KY465703.1 Lake Sinai virus 1 strain SA
KY465704.1 Lake Sinai virus 1 strain TAS
HQ871931.2 Lake Sinai virus 1
SRR801788 146978 length 208 cov 5.817680
SRR6901859 38187 length 156 cov 88.093023
SRR6901859 37878 length 4150 cov 104.525831
SRR801788 161659 length 263 cov 1.546610
SRR801844 70228 length 214 cov 1.556150
SRR6901859 37878 length 4150 cov 104.525831
SRR802538 56859 length 438 cov 2.053528
SRR801788 129107 length 197 cov 2.447059
SRR801788 30255 length 247 cov 2.654545
SRR802538 14256 length 351 cov 2.132716
SRR802538 57617 length 184 cov 2.229299
KY465717.1 Lake Sinai virus strain NT-LSV3
MG918125.1 Lake Sinai virus strain M92/2010 complete genome
SRR806693 2511 length 6036 cov 17.948078
SRR2032844 110349 length 6025 cov 11.412971
SRR2032857 70291 length 6040 cov 27.210544
KM886905.1 Lake Sinai virus strain exp10
KY354242.1 Lake Sinai Virus NE
SRR5109829 404803 length 5921 cov 17.656770
KM886902.1 Lake Sinai virus strain VBP022
KM886903.1 Lake Sinai virus strain VBP166
KX883223.1 Lake Sinai virus strain WHCC111282
SRR801788 146979 length 4476 cov 21.625084
KM886904.1 Lake Sinai virus strain VBP256
KY354243.1 Lake Sinai Virus SA1
KY354241.1 Lake Sinai Virus TO
SRR5109834 36661 length 5880 cov 19.973005
HQ888865.2 Lake Sinai virus 2 strain BruceSD T17E01
KY465706.1 Lake Sinai virus 2 strain WA1
KY465707.1 Lake Sinai virus 2 strain WA2
KY465710.1 Lake Sinai virus 2 strain VN3
KY465709.1 Lake Sinai virus 2 strain VN2
KY465712.1 Lake Sinai virus 2 strain TAS
KY465713.1 Lake Sinai virus 2 strain SA
KY465708.1 Lake Sinai virus 2 strain VN1
KY465711.1 Lake Sinai virus 2 strain QLD
KY465714.1 Lake Sinai virus strain WA2-LSV8
KY354244.1 Lake Sinai Virus SA2
KY465715.1 Lake Sinai virus strain VN2-LSV8
SRR2032817 153685 length 5778 cov 6.191097
LSV1
Sister1
LSV2
Sister2
0.050

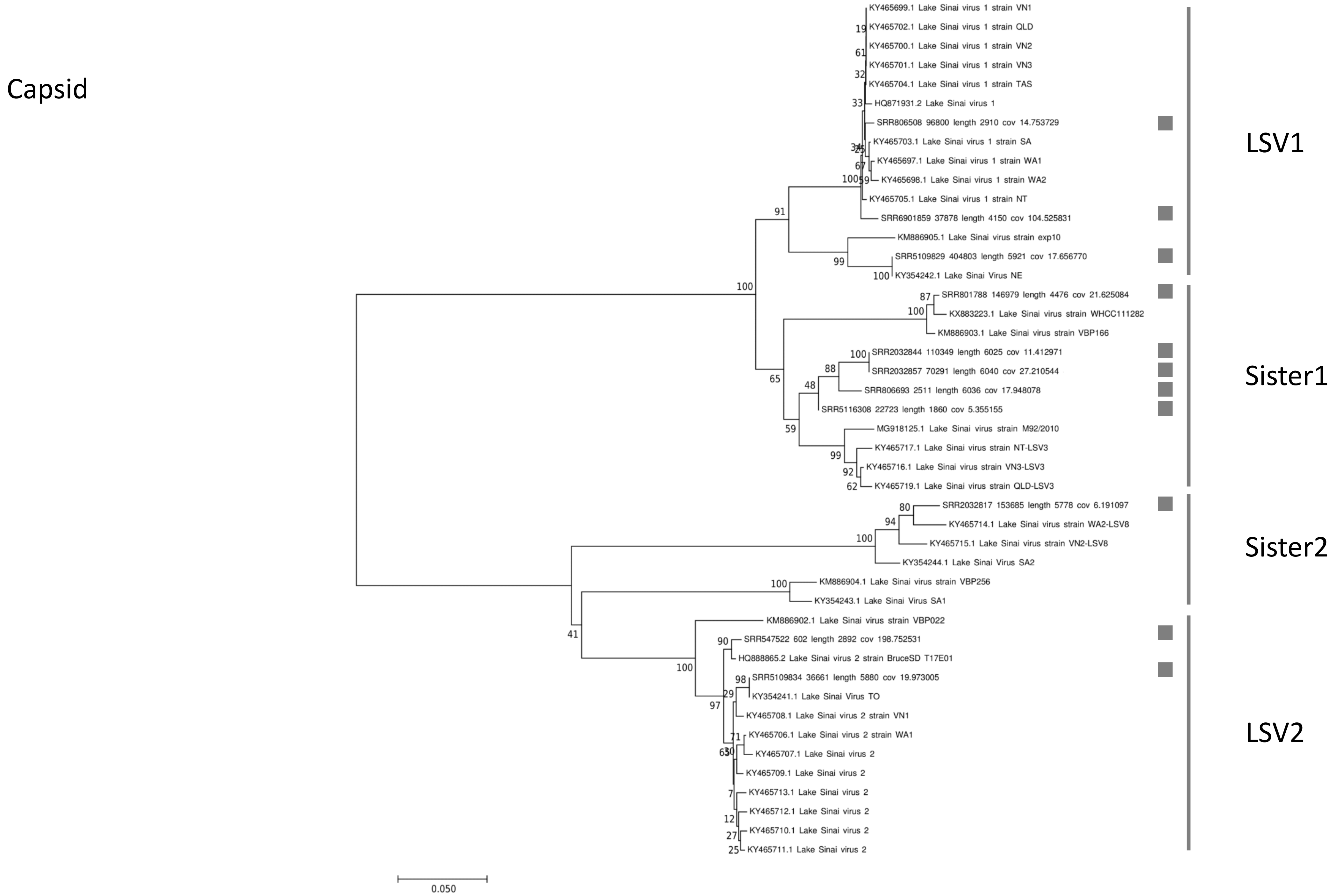
Capsid
KY465699.1 Lake Sinai virus 1 strain VN1
KY465702.1 Lake Sinai virus 1 strain QLD
KY465700.1 Lake Sinai virus 1 strain VN2
KY465701.1 Lake Sinai virus 1 strain VN3
KY465704.1 Lake Sinai virus 1 strain TAS
HQ871931.2 Lake Sinai virus 1
SRR806508 96800 length 2910 cov 14.753729
KY465703.1 Lake Sinai virus 1 strain SA
KY465697.1 Lake Sinai virus 1 strain WA1
KY465698.1 Lake Sinai virus 1 strain WA2
KY465705.1 Lake Sinai virus 1 strain NT
SRR6901859 37878 length 4150 cov 104.525831
KM886905.1 Lake Sinai virus strain exp10
SRR5109829 404803 length 5921 cov 17.656770
KY354242.1 Lake Sinai Virus NE
SRR801788 146979 length 4476 cov 21.625084
KX883223.1 Lake Sinai virus strain WHCC111282
KM886903.1 Lake Sinai virus strain VBP166
SRR2032844 110349 length 6025 cov 11.412971
SRR2032857 70291 length 6040 cov 27.210544
SRR806693 2511 length 6036 cov 17.948078
SRR5116308 22723 length 1860 cov 5.355155
MG918125.1 Lake Sinai virus strain M92/2010
KY465717.1 Lake Sinai virus strain NT-LSV3
KY465716.1 Lake Sinai virus strain VN3-LSV3
KY465719.1 Lake Sinai virus strain QLD-LSV3
SRR2032817 153685 length 5778 cov 6.191097
KY465714.1 Lake Sinai virus strain WA2-LSV8
KY465715.1 Lake Sinai virus strain VN2-LSV8
KY354244.1 Lake Sinai Virus SA2
KM886904.1 Lake Sinai virus strain VBP256
KY354243.1 Lake Sinai Virus SA1
KM886902.1 Lake Sinai virus strain VBP022
SRR547522 602 length 2892 cov 198.752531
HQ888865.2 Lake Sinai virus 2 strain BruceSD T17E01
SRR5109834 36661 length 5880 cov 19.973005
KY354241.1 Lake Sinai Virus TO
KY465708.1 Lake Sinai virus 2 strain VN1
KY465706.1 Lake Sinai virus 2 strain WA1
KY465707.1 Lake Sinai virus 2
KY465709.1 Lake Sinai virus 2
KY465713.1 Lake Sinai virus 2
KY465712.1 Lake Sinai virus 2
KY465710.1 Lake Sinai virus 2
KY465711.1 Lake Sinai virus 2
LSV1
Sister1
Sister2
LSV2
0.050

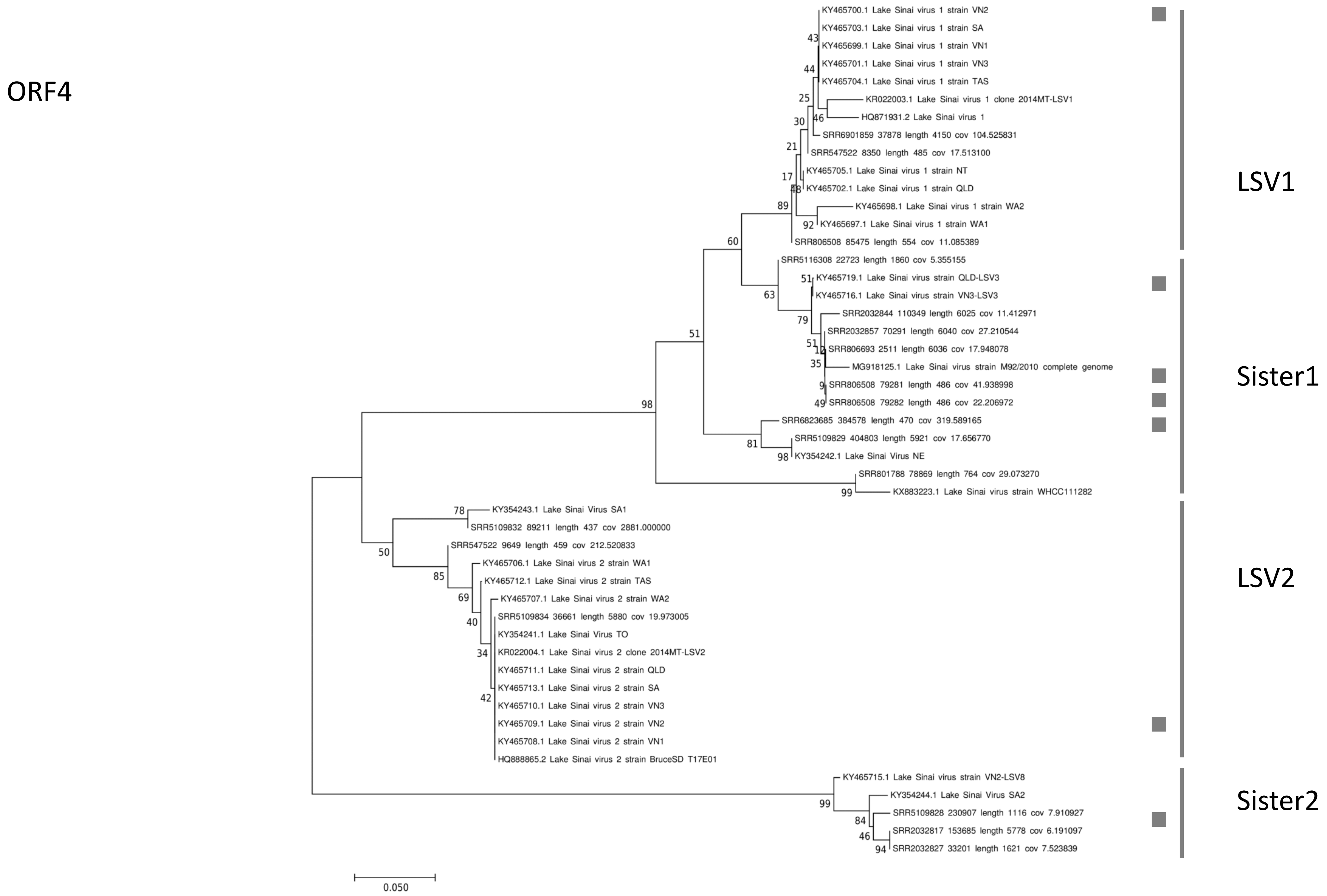
ORF4
KY465700.1 Lake Sinai virus 1 strain VN2
KY465703.1 Lake Sinai virus 1 strain SA
KY465699.1 Lake Sinai virus 1 strain VN1
KY465701.1 Lake Sinai virus 1 strain VN3
KY465704.1 Lake Sinai virus 1 strain TAS
KR022003.1 Lake Sinai virus 1 clone 2014MT-LSV1
HQ871931.2 Lake Sinai virus 1
SRR6901859 37878 length 4150 cov 104.525831
SRR547522 8350 length 485 cov 17.513100
KY465705.1 Lake Sinai virus 1 strain NT
KY465702.1 Lake Sinai virus 1 strain QLD
KY465698.1 Lake Sinai virus 1 strain WA2
KY465697.1 Lake Sinai virus 1 strain WA1
SRR806508 85475 length 554 cov 11.085389
SRR5116308 22723 length 1860 cov 5.355155
KY465719.1 Lake Sinai virus strain QLD-LSV3
KY465716.1 Lake Sinai virus strain VN3-LSV3
SRR2032844 110349 length 6025 cov 11.412971
SRR2032857 70291 length 6040 cov 27.210544
SRR806693 2511 length 6036 cov 17.948078
MG918125.1 Lake Sinai virus strain M92/2010 complete genome
SRR806508 79281 length 486 cov 41.938998
SRR806508 79282 length 486 cov 22.206972
SRR6823685 384578 length 470 cov 319.589165
SRR5109829 404803 length 5921 cov 17.656770
KY354242.1 Lake Sinai Virus NE
SRR801788 78869 length 764 cov 29.073270
KX883223.1 Lake Sinai virus strain WHCC111282
KY354243.1 Lake Sinai Virus SA1
SRR5109832 89211 length 437 cov 2881.000000
SRR547522 9649 length 459 cov 212.520833
KY465706.1 Lake Sinai virus 2 strain WA1
KY465712.1 Lake Sinai virus 2 strain TAS
KY465707.1 Lake Sinai virus 2 strain WA2
SRR5109834 36661 length 5880 cov 19.973005
KY354241.1 Lake Sinai Virus TO
KR022004.1 Lake Sinai virus 2 clone 2014MT-LSV2
KY465711.1 Lake Sinai virus 2 strain QLD
KY465713.1 Lake Sinai virus 2 strain SA
KY465710.1 Lake Sinai virus 2 strain VN3
KY465709.1 Lake Sinai virus 2 strain VN2
KY465708.1 Lake Sinai virus 2 strain VN1
HQ888865.2 Lake Sinai virus 2 strain BruceSD T17E01
KY465715.1 Lake Sinai virus strain VN2-LSV8
KY354244.1 Lake Sinai Virus SA2
SRR5109828 230907 length 1116 cov 7.910927
SRR2032817 153685 length 5778 cov 6.191097
SRR2032827 33201 length 1621 cov 7.523839
LSV1
Sister1
LSV2
Sister2
0.050
