# Supplementary figures and images for: Relative abundance and molecular evolution of Lake Sinai Virus (Sinaivirus) clades
Source: PeerJ. 2019 Mar 21;7:e6305. doi: 10.7717/peerj.6305 (PMC6431542; doi:10.7717/peerj.6305)

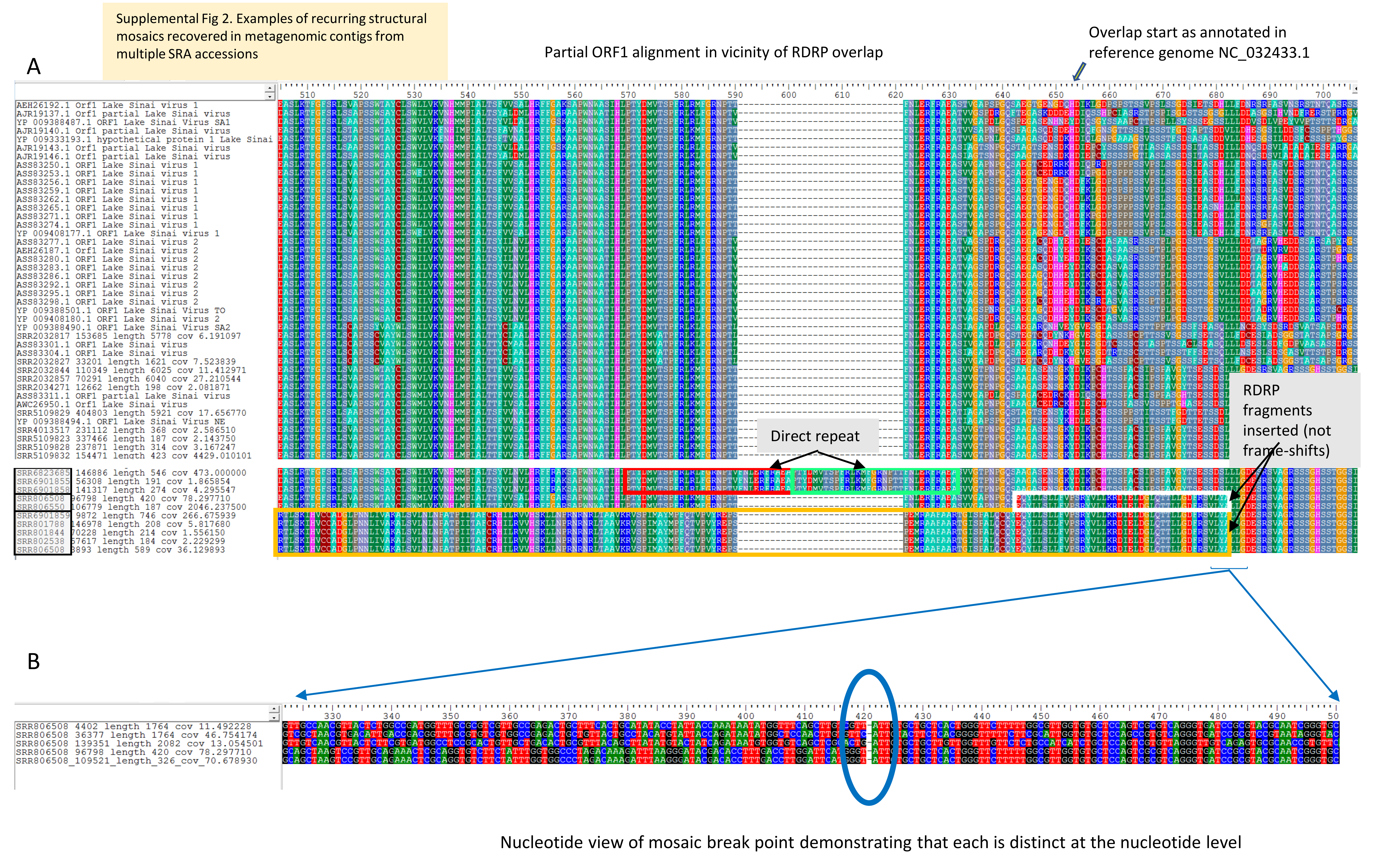

Supplement: Supplemental Information 6 [file peerj-07-6305-s006.png]
